# Effects of oral tauroursodeoxycholic acid and/or intestinal probiotics on serum biochemical indexes and bile composition in patients with cholecystolithiasis

**DOI:** 10.3389/fphar.2022.882764

**Published:** 2022-10-24

**Authors:** Fan Gao, Dongyao Guan, Gangliang Wang, Luting Zhang, Junmin He, Wenqiao Lv, Xiaofeng Zhang, Weifeng Tao, YeFeng Dai, Song Xu, Yeqi Chen, Bin Lu

**Affiliations:** Department of Hepatobiliary and Pancreatic Surgery, Shangyu People’s Hospital of Shaoxing, Shaoxing, China

**Keywords:** tauroursodeoxycholic acid, intestinal probiotics, cholecystolithiasis, bile composition, cholelithiasis

## Abstract

**Background:** In recent years, gallstones have become a major condition affecting people’s health. Cholecystectomy remains an effective treatment method, but it has large risk factors. It is well known that the hepatoenteric axis plays a key role in gallstone formation, and it is gradually becoming a research focus. Cholesterol homeostasis can be regulated by the liver and intestinal tract in our bodies, and intestinal flora can regulate the digestion and absorption of cholesterol. These two factors are closely related to the formation of gallstones.

**Aim:** To investigate the effects of tauroursodeoxycholic acid (TUDCA) and/or intestinal probiotics on serum biochemical indexes and bile composition in patients with cholecystolithiasis.

**Methods:** For this study, 96 patients with cholecystolithiasis were recruited at our hospital. The patients were randomly divided into four groups according to a random number table: group Ⅰ (TUDCA, 24 cases), group Ⅱ (intestinal probiotics, 24 cases), group Ⅲ (TUDCA and intestinal probiotics, 24 cases) and group Ⅳ (control group, 24 cases). All patients underwent laparoscopic gallbladder-preserving lithotomy or laparoscopic cholecystectomy. Bile samples were identified and extracted during the operation.

**Results:** The results revealed that the levels of serum total bile acid (TBA), serum total cholesterol (TCHOL) and serum triglyceride in groups I, II and III before and after the intervention were statistically significant (*p* < 0.05). There were significant differences in serum low-density lipoprotein cholesterol (LDL-C) between groups I and II before and after the intervention (*p* < 0.05), but the serum LDL-C level in group Ⅲ before and after the intervention was similar (*p* > 0.05). Regarding bile, TBA levels demonstrated no significant difference between groups I and III (*p* > 0.05), and the differences between the other two groups were statistically significant (*p* < 0.05). No significant difference was identified in phospholipid and TCHOL levels between groups I and Ⅲ (*p* > 0.05), and the differences between the other two groups were statistically significant (*p* < 0.05). There were significant differences in the levels of free Ca^2+^, pH value and glycoprotein in bile among the four groups (*p* < 0.05). The levels of cholic acid, chenodeoxycholic acid and deoxycholic acid in bile were significantly different among the four groups (*p* < 0.05). The level of lithocholic acid (LCA) in groups Ⅱ and Ⅲ was similar, as was the level of LCA in groups I and ⅠV, but the difference in level between the other two groups was statistically significant (*p* < 0.05).

**Conclusion:** The combination of TUDCA and intestinal probiotics did not enhance the effect of either treatment. The use of intestinal probiotics alone can maximise the reverse development of bile composition in patients with cholecystolithiasis compared with TUDCA alone and a combination of TUDCA and intestinal probiotics, thereby reducing gallstone formation.

## Introduction

Cholelithiasis, also known as gallstones, is a common disease in the hepatobiliary surgery field. According to their composition, gallstones can be divided into cholesterol stones, pigment stones and mixed stones (Chandran et al., 2007) and can be further divided into cholecystolithiasis and choledocholithiasis depending on their location. In East Asia, the incidence of brown bile pigment stones in the bile duct is relatively high and may cause devastating cholangitis (Lambou-Gianoukos and Heller, 2008). In China, the morbidity of cholelithiasis is 7%–10% in adults (Qiao et al., 2017). Moreover, because of an increase in the prevalence of obesity and acceleration of the ageing process in the population, the occurrence of cholelithiasis may increase further in the near future (Venneman and van Erpecum, 2010). Therefore, the prevention of gallstone formation should be the focus of future research. The hepatic hypersecretion of cholesterol is a risk factor of cholelithiasis (Di Ciaula et al., 2018). The intestinal flora also provides a novel link to the pathogenesis of cholelithiasis (Grigor’eva, 2020).

Cholesterol homeostasis plays a critical role in maintaining normal cell and system function (Luo et al., 2020), and homeostasis is mainly regulated by the liver and intestinal tract in our bodies (Zhao and Dahlman-Wright, 2010). Bile acids (BAs) are cholesterol derivatives that act as signal molecules by activating nuclear and G protein-coupled receptors (Van den Bossche et al., 2017a). The activation of these receptors changes the expression of genes involved in different processes, including BA homeostasis, lipid metabolism and inflammation (Copple and Li, 2016). Some randomised clinical trials were conducted in the 1990s and 2000s, revealing that gallstone formation was significantly reduced in ursodeoxycholic acid (UDCA) groups compared with placebo groups (Williams et al., 1993; Sugerman et al., 1995; Miller et al., 2003). At least one of the UDCA accelerating BA enterohepatic circulation mechanisms has been found to accelerate enterohepatic circulation by inhibiting the intestinal farnesoid X receptor (FXR) signalling pathway (Sun et al., 2018). Tauroursodeoxycholic acid (TUDCA), a cytoprotective secondary BA derived from UDCA (Amaral et al., 2016), is a more effective inhibitor of this pathway in intestinal cells *in vitro* (Sun et al., 2018), and it is produced in the liver by conjugation of taurine to ursodeoxycholic acid (UDCA). In addition, there is evidence that TUDCA can inhibit the absorption and synthesis of lipids in the small intestine by improving the intestinal flora in mice fed a high-fat diet, thus reducing gallstone formation (Lu et al., 2021). Studies have shown that the bile acid TUDCA can cross the blood-brain barrier and have a low toxicity profile (Cortez and Sim, 2014; Vang et al., 2014).

Intestinal flora play a key role in the formation of cholelithiasis (Chen et al., 2021), not only by regulating digestion and absorbing cholesterol but also by influencing the metabolism of BAs (Wang and Xue, 2020). Lactic acid bacteria are Gram-positive bacteria, so called lactic acid bacteria because they can ferment carbohydrates to produce lactic acid. Lactobacillus is a kind of lactic acid bacteria, and as one of the normal flora of human gut, it plays an important role in maintaining the stability of intestinal microecology (Quan and Xing, 2019). Cholesterol synthesises primary BAs in the liver that have been expelled from the intestine in response to food. Gut microbiota promote the deconjugation and biotransformation of primary BAs into secondary BAs through bile salt hydrolase (BSH), which is produced intestinally, and are then reabsorbed in the terminal ileum and colon and returned to the liver through the portal vein (Jiang et al., 2017). The main physiological functions of BAs are the digestion and absorption of intestinal cholesterol, regulatory hepatic biosynthesis and gallbladder motor function. BAs also modulate lipid and glucose metabolisms by activating FXR in the liver and intestine (Ridlon et al., 2006; Di Ciaula et al., 2017). In addition, BSH hydrolyses the amide bond between bile salt and its conjugated amino acid (C-24N-acylamide) to release glycine or taurine from steroid nuclei, which are then referred to as unconjugated bile salts. In deconjugates, the solubility of bile salts is low, and the amount of bile salt absorbed by intestinal cells is lower than that of conjugates. These salts are then excreted in faeces. As the excretion of bile salt in faeces increases, less is returned to the liver through enterohepatic circulation, which increases the demand for cholesterol, prompting the liver to synthesise bile salt. Therefore, the liver increases the expression of low-density lipoprotein cholesterol (LDL-C) receptors, thus increasing the liver’s uptake of LDL-C from circulation and decreasing the concentration of serum LDL-C and total cholesterol (TC) (Khan et al., 2009; Wahlström et al., 2017). The liver regulates the synthesis of cholesterol and the production of bile salts, and the intestine regulates the absorption of BAs and the excretion of bile salts. Therefore, regulating cholesterol in human blood by treating the liver and intestines may be an effective way of preventing the occurrence of gallstones. To better demonstrate this conjecture, we conducted this study, recruiting patients with cholecystolithiasis as the primary research participants and exploring the effects of oral TUDCA and/or intestinal probiotics on serum biochemical indexes and bile composition among patients with cholecystolithiasis.

## Patients and methods

### Study participants

In this study, the participants were selected from patients with cholecystolithiasis who visited our hospital between June 2020 and December 2020. All 96 patients who met the criteria were included, and the index changes in these patients were observed and recorded.

The patients were randomly divided into four groups according to a random number table: group Ⅰ (TUDCA, 24 cases), group Ⅱ (intestinal probiotics, 24 cases), group Ⅲ (TUDCA and intestinal probiotics, 24 cases) and group Ⅳ (control group, 24 cases). All patients underwent laparoscopic gallbladder-preserving lithotomy or laparoscopic cholecystectomy. Bile samples were identified and extracted during the operation. In this study, the diagnosis of gallstones was based on a B-mode ultrasound. This study was conducted in accordance with the Declaration of Helsinki and approved by the Ethics Committee of our hospital. All participants signed an informed consent form.

### Inclusion and exclusion criteria

Inclusion criteria: 1) a diagnosis of gallstones using ultrasound and no intrahepatic or extrahepatic bile duct stones; 2) a preoperative ultrasound gallbladder fatty meal test demonstrating that the maximum contraction rate of the gallbladder was ≥30%; 3) no history of acute cholecystitis within 3 months; 4) a gallbladder that had a normal shape and size and no stone obstruction in the gallbladder duct; 5) able to eat three meals a day as directed; 6) no history of abdominal operation, normal liver function, and no life-threatening complications; 7) a signed informed consent form.

Exclusion criteria: 1) intrahepatic and extrahepatic choledocholithiasis, history of gastrointestinal surgery or pancreatitis; 2) acute inflammation of the gallbladder and a suspected or diagnosed malignant gallbladder tumour; 3) confinement of gallbladder duct stones; 4) refusal to take oral drugs or drugs taken irregularly before the operation; 5) an irregular diet; 6) initially diagnosed as pigment cholelithiasis during the operation; 7) use of antibiotics before the operation.

### Sample size evaluation

The formula for estimating the sample size: *N = Z* ^*2*^ *×* (*P ×* (1—*P*))*/E* ^*2*^ (*N*: sample size; *Z* = 1.64%; *E* = 0.1; *p* = 10%, and *P* is the incidence of cholecystolithiasis). Therefore, *N* was 24 cases according to the above formula.

## Material and methods

After all the participants had completed the admission procedures, the relevant auxiliary and serum biochemical examinations were performed. Following the preoperative examination, the feasibility of the operation was evaluated by the surgeon, and the anaesthesiologist evaluated the risk of the operation. Laparoscopic gallbladder-preserving lithotomy or laparoscopic cholecystectomy was performed after excluding surgical restrictions. All participants received a serum biochemical examination before the operation, and bile was extracted during the operation.

Patients in the control group (group IV) were not treated with a drug intervention. The intervention measures of the other three groups were as follows. 1) Patients in group Ⅰ received oral TUDCA (5–10 mg/kg/d; Taurolite, Bruschettini, Italy). The duration of the study treatment was 2 weeks. The dose was two tablets per night for patients weighing less than 75 kg or three tablets per night for patients weighing more than 75 kg. 2) Patients in group Ⅱ took compound *Lactobacillus acidophilus* tablets (5 × 10^6^ *L. acidophilus*/tablet; Yi jun kang, Tonghua, China) for 2 weeks. The dose for patients weighing less than 75 kg was two tablets at a time, and for those of more than 75 kg it was three tablets at a time. 3) Patients in group Ⅲ were treated with TUDCA combined with compound *L. acidophilus* tablets (the dose was similar to groups I and II) for 2 weeks.

### Main observation indicators

In this study, the leading observation indicators were the levels of total bile acid (TBA), total cholesterol (TCHOL), triglyceride (TG), LDL-C, phospholipid (PL), glycoprotein, free Ca^2+^, pH value, cholic acid (CA), chenodeoxycholic acid (CDCA), deoxycholic acid (DCA) and lithocholic acid (LCA) in the bile. These indicators have a certain relationship with the formation of gallstones.

### Sample collection methods

The laboratory technicians in our hospital collected all the serum samples in this study. To collect the bile, the gallbladder triangle was carefully separated under laparoscopy to identify the cystic duct during surgery. A 5.5 paediatric scalp needle (Shandong/Saihua) was then inserted into the abdominal cavity through the main operation incision and carefully inserted into the cystic duct and straight into the common bile duct in the direction of the cystic duct. The bile was slowly extracted by a nurse with an external 10-ml syringe. During this process, a clamp was attached to the scalp needle to avoid damaging the surrounding tissue. After the bile extraction, the scalp needle was carefully removed from the body, and the rest of the operation was performed. If laparoscopic cholecystectomy was performed, the cystic duct was ligated directly with a biological clip; if laparoscopic gallbladder-preserving lithotomy was performed, the puncture point was observed again after the completion of the operation. If there was no bile leakage, the abdomen was closed; otherwise, 3-0 Xiekang sutures were used to suture the puncture point. After the operation, the extracted bile samples were packed into three 1.5-ml Eppendorf tubes and sent to the central laboratory of Shangyu People’s Hospital of Shaoxing within 30 min for storage at −80°C. If the central laboratory was closed, the samples were temporarily stored in a −20°C freezer and then transferred to the −80°C freezer when the laboratory opened. Each bile specimen was marked with a serial number according to the requirements, and general information (name, sex, age, hospitalisation number and diagnosis) was recorded in the scientific research record book along with the operation conditions (e.g., operation mode, operation duration, intraoperative blood loss, stone condition and bile character), postoperative recovery condition (hospitalisation time and complications after surgery) and follow-up after discharge.

### Detection methods

In this study, the levels of TBA, TCHOL, TG and LDL-C in the serum biochemical examination indexes were detected using a C16000 Biochemical Analyzer (Abbott, United States) in the Department of Biochemistry of Shangyu People’s Hospital of Shaoxing. The levels of TBA, PL and TCHOL in the bile were detected using an automatic biochemical analyser in the central laboratory of Kunming Medical University (Shenzhen Mindray Bio-medical Electronics, Shenzhen, China). Concentrations of Ca^2+^ and pH in the bile were detected using an Orion Dual StarTM ion/pH instrument (ThermoFisher, United States). The glycoprotein levels in the bile were determined using the amino hexose method with an ultraviolet spectrophotometer (Shanghai Hewlett-Packard Analytical Instrument, Shanghai, China). The levels of CA, CDCA, DCA, and LCA in the bile were integrated through liquid chromatography (Waters ACQUITY) and mass spectrometry (ABSCIEX5500QQQ-MS) using MultiQuant software (Frankenberg et al., 2006), and levels were calculated using a standard curve.

### Statistical analysis

In this study, SPSS 21.0 software (IBM, Chicago, IL, United States) was used for statistical analysis, the measurement data were expressed as mean ± standard deviation ( $\bar{\text{x}}$ ± SD) and an independent sample *t*-test was used. The enumeration data were analysed using a χ2 test or Fisher’s exact test. A value of *p* < 0.05 was considered statistically significant.

## Results

### General characteristics

A total of 96 patients with cholecystolithiasis were included in this study and were divided into four groups: group Ⅰ (TUDCA, 24 cases), group Ⅱ (intestinal probiotics, 24 cases), group Ⅲ (TUDCA and intestinal probiotics, 24 cases) and group Ⅳ (control group, 24 cases). There were no significant differences in sex, age or mode of operation among the patients in each group (*p* > 0.05) (Table 1).

**TABLE 1:** The general characteristics of each group of patients.

Clinical features	groupⅠ	groupⅡ	groupⅢ	groupⅣ	*p* Value
sex(Number)					
Male	5	6	9	8	>0.05
Female	19	18	15	16	>0.05
Age(Years old)	48.1 ± 13.8	45.4 ± 14.4	47.6 ± 16.9	43.5 ± 15.7	>0.05
mode of operation (Number)					
LC	17	17	12	13	>0.05
LRCL	7	7	12	11	>0.05

LC: laparoscopic cholecystectomy. LRCL: Laparoscopic choledochoscope combined with minimally invasive gallbladder-preserving lithotomy.

### Levels of serum biochemical indexes

The change in three serum biochemical indexes (TBA, TCHOL, and TG) were significant in all three groups. In group I, four indicators, including LDL-C (mmol/L), were significant. However, the changes in LDL-C (mmol/L) in groups II and III were nonsignificant. After the intervention, TBA increased in group I, and the other three indexes (TCHOL, TG, and LDL-C) exhibited a significant decrease; however, in groups II and III, the four indexes all decreased. The details are presented in Table 2.

**TABLE 2:** Comparison the level of serum biochemical indexes of different groups.

Intervention	Before	After	*p* Value
Group Ⅰ			
TBA (μmol/L)	1.42 ± 0.49	2.20 ± 1.27*	<0.05
TCHOL (mmol/L)	5.30 ± 0.18	4.68 ± 0.40***	<0.001
TG (mmol/L)	2.33 ± 0.60	1.43 ± 0.57***	<0.001
LDL-C(mmol/L)	3.27 ± 0.34	2.92 ± 0.31***	<0.001
Group Ⅱ			
TBA (μmol/L)	5.22 ± 2.66	1.36 ± 0.78***	<0.001
TCHOL (mmol/L)	4.32 ± 0.84	3.61 ± 0.98*	0.010
TG (mmol/L)	1.89 ± 1.05	0.87 ± 0.35***	<0.001
LDL-C (mmol/L)	2.48 ± 0.48	2.09 ± 0.68*	0.026
Group Ⅲ			
TBA (μmol/L)	3.87 ± 1.36	1.50 ± 1.06***	<0.001
TCHOL (mmol/L)	4.67 ± 1.19	3.99 ± 1.06**	0.004
TG (mmol/L)	1.54 ± 0.72	1.14 ± 0.51**	0.003
LDL-C (mmol/L)	2.86 ± 1.10	2.43 ± 0.91	0.147

*p* < 0.05 was considered to be statistically significant. Compared with the before intervention, *p < 0.05. TBA, total bile acid; TCHOL, serum total cholesterol; TG, triglyceride; LDL-C, low-density lipoprotein cholesterol.

### Levels of total bile acid, phospholipid and total cholesterol

**FIGURE 1:**
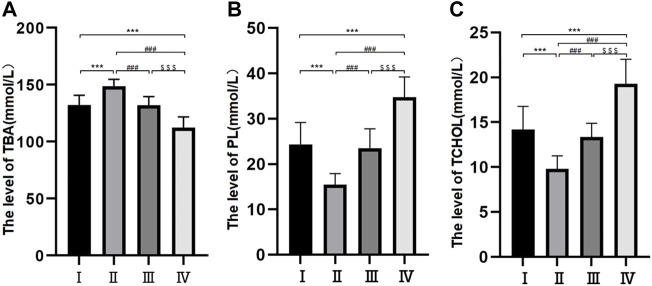
Bile composition histogram of TBA, PL and TCHOL. *p < 0.05, **p < 0.01, ***p < 0.001 versus group I; #*p* < 0.05, ##*p* < 0.01, ###*p* < 0.001 versus group II. $ *p* < 0.05, $$ *p* < 0.01, $$$ *p* < 0.001 versus group III. Samples were collected during the patient’s operation. The levels of TBA, PL, and TCHOL in the four groups were all different and demonstrated significant differences between different groups except for groups Ⅰ and Ⅲ (*p* < 0.05).

Samples were collected during the patient’s operation. The levels of TBA in the four groups were all different and demonstrated significant differences between different groups except for groups Ⅰ and Ⅲ (*p* < 0.05) (Figure 1A). Samples were collected during the patient’s operation. The levels of PL in the four groups were all different and demonstrated significant differences between different groups except for groups Ⅰ and Ⅲ (*p* < 0.05) (Figure 1B). Samples were collected during the patient’s operation. The levels of TCHOL in the four groups were all different and demonstrated significant differences between different groups except for groups Ⅰ and Ⅲ (*p* < 0.05) (Figure 1C).

### Levels of Ca^2+^ and pH in the bile

**FIGURE 2:**
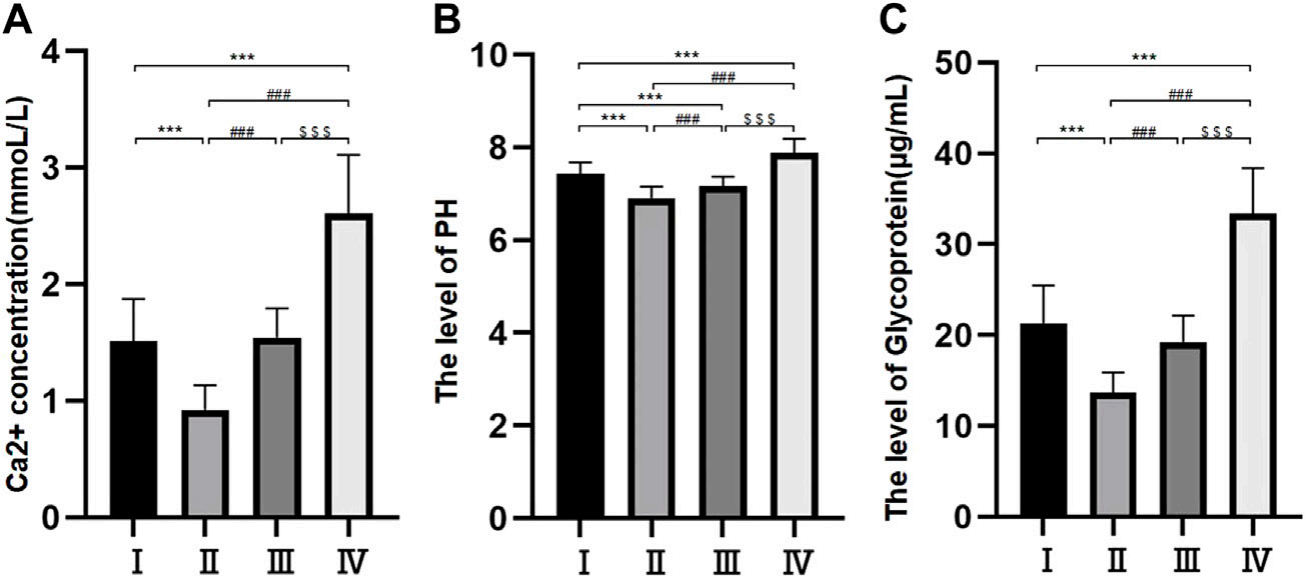
Histogram of free Ca2+ concentration in bile components **(A)**; Bile pH histogram **(B)**; Histogram of bile glycoprotein level **(C)**. *p < 0.05, **p < 0.01, ***p < 0.001 versus group I; #*p* < 0.05, ##*p* < 0.01, ###*p* < 0.001 versus group II. $ *p* < 0.05, $$ *p* < 0.01, $$$ *p* < 0.001 versus group III.

The levels of Ca^2+^ and pH were different among the different groups. The levels of Ca^2+^ were 0.92 ± 0.21 mmol/L in group Ⅱ, 1.51 ± 0.36 mmol/L in group I, 1.54 ± 0.25 mmol/L in group Ⅲ and 2.61 ± 0.50 mmol/L in group Ⅳ. The difference between groups I and II was nonsignificant, but the differences among the other three groups were all significant (*p* < 0.05) (Figure 2A). The pH levels were 6.9 ± 0.25 mmol/L in group Ⅱ, 7.16 ± 0.21 mmol/L in group Ⅲ, 7.43 ± 0.25 mmol/L in group I and 7.88 ± 0.31 mmol/L in group Ⅳ. The differences among the four groups were statistically significant (*p* < 0.05) (Figure 2B).

### Levels of glycoprotein in the bile

The levels of glycoprotein in the bile were 13.69 ± 2.21 μg/ml in group Ⅱ, 19.21 ± 2.93 μg/ml in group Ⅲ, 21.31 ± 4.17 μg/ml in group Ⅰ and 33.39 ± 5.02 μg/ml in group Ⅳ. The differences among the four groups were statistically significant (*p* < 0.05) (Figure 2C).

### Levels of cholic acid, chenodeoxycholic acid, deoxycholic acid and lithocholic acid

**FIGURE 3:**
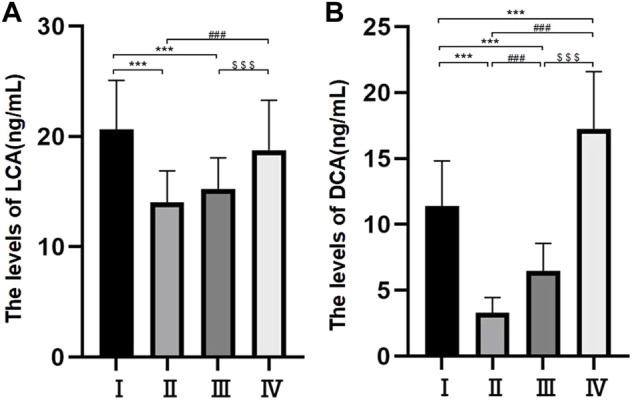
Bile composition histogram of LCA **(A)**. Bile composition histogram of DCA **(B)** *p < 0.05, **p < 0.01, ***p < 0.001 versus group I #*p* < 0.05, ##*p* < 0.01, ###*p* < 0.001 versus group II. $ *p* < 0.05, $$ *p* < 0.01, $$$ *p* < 0.001 versus group III.

Differences in the levels of CA, CDCA and DCA among the four groups were statistically significant (*p* < 0.05) (Table 3 and Figure 3A). The levels of LCA were 14.04 ± 2.86 ng/ml in group Ⅱ, 15.26 ± 2.81 ng/ml in group Ⅲ, 18.75 ± 4.53 ng/ml in group Ⅳ and 20.66 ± 4.43 ng/ml in group Ⅰ. The differences between groups Ⅱ and Ⅲ and groups Ⅳ and Ⅰ were not statistically significant, but the difference between the other two groups was statistically significant. The levels of DCA were 3.29 ± 1.16 ng/ml in group Ⅱ, 6.49 ± 2.06 ng/ml in group Ⅲ, 11.41 ± 3.41 ng/ml in group Ⅰ and 17.25 ± 4.35 ng/ml in group Ⅳ, and there were statistically significant differences among the four groups (*p* < 0.05) (Figure 3B).

**TABLE 3:** The level of bile acids of different groups.

Groups	Ⅰ	Ⅱ	Ⅲ	Ⅳ
Bile acids				
CA(ng/ml)	142.46 ± 43.21	29.16 ± 10.19	58.18 ± 16.87	89.55 ± 26.36
CDCA(ng/ml)	11.29 ± 3.29	3.60 ± 1.05	6.98 ± 2.11	17.93 ± 4.47

CA, Cholic acid. CDCA, chenodeoxycholic acid.

## Discussion

In this study, we acquired bile and serum from a cohort of 96 patients with cholecystolithiasis, 24 of whom formed a non-drug control group and 72 drug intervention groups. Changes in the patients’ serum biochemical indexes were identified before and after the intervention and bile components were detected after bile extraction during the operation. All possible factors leading to the formation of gallstones were comprehensively considered, demonstrating that intestinal flora may affect BA metabolism and cause cholelithiasis (Tang et al., 2017). The oral administration of intestinal probiotics alone significantly reduced serum TBA, TCHOL, TG and LDL-C when compared with the administration of TUDCA, but the combination of these two drugs did not produce any synergistic effects. After the bile was extracted from patients, the levels of TBA were the highest and the levels of PL, TCHOL, Ca^2+^, pH value, glycoprotein, CA, CDCA, DCA, and LCA were the lowest in the intestinal probiotics group.

Generally, cholesterol synthesises conjugated BA under the action of 7 α-hydroxylase. The conjugated BA, which is discharged into the intestine, then becomes free BA and is finally absorbed by the intestinal epithelial cells. In this process, intestinal microflora with BSH activity regulates BA synthesis by controlling the gene expression level of 7 α-hydroxylase (Song et al., 2019), which is a rate-limiting enzyme and also a key enzyme in cholesterol elimination (Fiorucci and Distrutti, 2015). Free BAs (CA, CDCA, DCA, and LCA) act as key signal molecules in the activation of FXR, inhibit the promotion of 7 α-hydroxylase, downregulate the gene expression of 7 α-hydroxylase and reduce the synthesis of BAs (Kliewer and Mangelsdorf, 2015). The aforementioned intestinal flora uncouples the binding BAs to form free BAs. However, after intestinal microbiota colonisation in sterile mice with cholelithiasis, it was found that the level of the FXR antagonist T-β-MCA (taurine-bound β-mouse cholic acid) decreased significantly, increasing FXR expression and decreasing BA synthesis (Wu et al., 2013). Among patients prone to gallstones, an imbalance in intestinal flora leads to the enhancement of BSH activity, and excessive free BA in the intestine initiates a negative feedback regulation mechanism that inhibits the synthesis of BA. This long-term vicious circle leads to decreased BA, the oversaturation of cholesterol and the precipitation of stones in bile.

In addition, 7 α-dihydroxy bacteria rely on BA 3 α-hydroxysteroid dehydrogenase, BA coenzyme A ligase and BA 7 α-dehydratase (Ridlon et al., 2020). At present, only some strains of *Clostridium*, *Lachnospiraceae* and *Peptostreptococcaceae* are known to have 7 α-dehydroxylation activity. Although they account for a small part of the whole intestinal bacteria (<1%), they can have a significant impact on the host and increase the secondary BA DCA and LCA in the intestine. DCA and LCA have also been found to inhibit FXR in the presence of CDCA (Jiao et al., 2018; Chen et al., 2019). Thus, the potential impact of intestinal flora on the pathogenesis of cholesterol gallstones cannot be ignored (Van den Bossche et al., 2017b; Wu et al., 2020).

In the present study, we investigated a number of patients with cholecystolithiasis treated with oral administered intestinal probiotics and TUDCA. Correlations between the oral drug group, cholecystolithiasis and laboratory test indices were also evaluated. Therefore, our results may reflect causality between the medicines and disease, providing a theoretical basis for the development of oral intestinal probiotic-driven cholecystolithiasis treatments in the future. In addition, the research on the treatment of cholelithiasis with traditional Chinese medicine is also increasing, which also has a potential in the future.

TUDCA and UDCA can both dissolve cholesterol gallstones effectively (Zhao et al., 2016; Zhang et al., 2019). Although there is no substantial evidence that intestinal probiotics can dissolve gallbladder stones, our research revealed that serum biochemical indexes (TBA, TCHOL, LDL-C, and TG) in patients with cholecystolithiasis were significantly decreased after treatment with TUDCA and/or intestinal probiotics, especially intestinal probiotics. These results indicate that our intervention had a positive effect on inhibiting gallstones. Moreover, intestinal flora may affect BA metabolism (Tang et al., 2017), and there is evidence that TUDCA, as a representative of hydrophilic BAs, can reduce the absorption of cholesterol in the small intestine (de Vogel-van den Bosch et al., 2008). Therefore, we believe that intestinal probiotics may be an effective therapeutic drug for inhibiting gallstones. In addition, studies have shown that bile with pH < 7.1 is beneficial to the dissolution of calcium salt (Hofmann and Mysels, 1992), and intestinal probiotics can reduce pH to below 7.1, which is consistent with the literature. In our study, we demonstrated that the levels of glycoprotein and pH value in the intervention groups were significantly lower than those in the control group. Therefore, the oral administration of intestinal probiotics alone may be superior for reducing the concentrations of glycoprotein, Ca^2+^ and pH in bile. In addition, our study revealed that DCA and LCA can produce gallstones (Palmer, 1965; Tazuma et al., 2017), whereas the levels of DCA and LCA in the drug adding group decreased in our study, suggesting that oral intestinal probiotics alone can effectively reduce these two indicators in the bile of patients with gallstones. Furthermore, we noted that the level of TCHOL in the bile of patients in the three observation groups after the intervention was lower than that of the control group; the level of TBA was higher than that of the control group, and the difference was statistically significant. These results demonstrate that changes in TCHOL and TBA in the bile components can develop in the opposite direction of cholecystolithiasis, with the largest change in group Ⅱ. The changing trend in PL is not in line with expectations, and further research is required to identify the reasons. In addition, in this study, the differences in these three indexes between groups Ⅰ and Ⅲ were not statistically significant, indicating that the combined use of the two drugs does not enhance the effect of TUDCA alone but weakens the effect of the intestinal probiotics alone. However, the specific mechanism remains undetermined.

## Advantages and limitations

First, in this study, intestinal probiotics were introduced as an intervention measure to change the bile composition of patients with cholecystolithiasis. The comparison between intestinal probiotics and TUDCA has rarely been reported in the literature. Second, a 5.5 paediatric scalp needle was used to extract bile from the common bile duct through the cystic duct under laparoscopy. This is different from previous studies, which extracted gallbladder bile through the gallbladder wall; this a further point of innovation. This operation can largely avoid the inaccurate results caused by the change in bile composition resulting from the concentration and secretion of gallbladder bile through the gallbladder wall, although the operation process is more complex and still has certain risks. However, there are still several limitations to this study. First, errors caused by a limited sample size and individual differences are inevitable. Second, bile extraction can only be performed once during an operation, and the samples cannot be compared before and after the intervention. Therefore, errors caused by differences between groups cannot be excluded. Third, because of the significant difference in the formation mechanism of cholesterol stones and gallstones, the effects of the intervention measures on these two types of stones remain unclear. In addition, we did not explore whether there is a potential impact on the composition of the patient’s own microbiota in the treatment of patients with TUDCA +/− probiotics.

## Conclusion

In conclusion, TUDCA alone can reduce serum TCHOL, TG and LDL-C levels and increase TBA levels in patients with cholecystolithiasis. Intestinal probiotics alone can reduce serum TBA, TCHOL, TG, and LDL-C levels. The combination of TUDCA and intestinal probiotics did not enhance the effect of TUDCA or intestinal probiotics, but the specific mechanism is still worthy of further exploration. The use of intestinal probiotics alone may maximise the reverse development of bile composition in patients with cholecystolithiasis compared with TUDCA alone and a combination of TUDCA and intestinal probiotics, thereby reducing gallstone formation. Besides, it is also worth considering whether the composition of the patient’s own microbiota could potentially influence the outcome when treating patients with TUDCA +/− probiotics.

## Data Availability

The original contributions presented in the study are included in the article/Supplementary Material, further inquiries can be directed to the corresponding author.
